# Can we predict femoral head vitality during surgical hip dislocation?

**DOI:** 10.1093/jhps/hnu010

**Published:** 2014-10-21

**Authors:** Alessandro Aprato, Andrea Bonani, Matteo Giachino, Marco Favuto, Francesco Atzori, Alessandro Masse′

**Affiliations:** 1. Orthopaedic Department, School of Medicine, San Luigi Hospital, Regione Gonzole 10, 10043 Orbassano, TO; 2. School of Medicine, University of Turin, C.so Massimo d'Azeglio 60, 10126 Torino, Italy

## Abstract

**Purpose:** Surgical hip dislocation is commonly performed in orthopaedic surgery for several pathologies that often present risk of avascular necrosis (AVN) of femoral head. Observation of blood spilling out from a drill hole, performed in the head after dislocation, has been proposed as a predictive test for AVN. No data have been published about test reliability. Study’s aim was to evaluate the correlation between ‘bleeding sign’ and AVN in surgical dislocation for elective disease and for acetabular fractures.

**Methods:** All patients meeting the indication for surgical dislocation were included in this prospective study. Patients with follow-up shorter than 8 months were excluded. Intra-operative assessment of head vascularity was performed in 44 patients through the ‘bleeding sign’: a 2.0-mm drill hole carried out on the head during surgery. A positive bleeding test was considered an immediate appearance of active bleeding. Development of AVN was considered the main outcome. Necrosis group criteria were detection of type II, III or IV X-ray according to Ficat classification.

**Results:** Forty-four patients with selected acetabular fractures, slipped capital femoral epiphysis and femoral head deformity were enrolled. Mean age was 25 years and mean follow-up was 36 months. Thirty-eight patients presented positive intra-operative bleeding sign and six demonstrated no bleeding. Sensitivity for the ‘bleeding sign’ was 97%, specificity was 83%, positive predictive value was 97%, negative predictive value was 83% and accuracy was 95% (*P* < 0.001).

**Conclusions:** Bleeding after head drilling is a reliable test for AVN in patients who undergo a surgical hip dislocation.

## INTRODUCTION

Surgical hip dislocation has become a widely performed technique both for elective and traumatic surgery [1–6]. Surgical hip dislocation is also called ‘safe dislocation’ because its technique avoids avascular necrosis (AVN) by protecting the vessels of the femoral head during surgery. On the other hand, AVN is the most unfavorable complication of several pathologies treated with this technique [e.g., slipped capital femoral epiphysis (SCFE), severe femoral head disorders and acetabular fractures], leading, if not treated, to progressive sub-chondral bone collapse, deformities and eventually complete degeneration of the joint [7–11].

Blood supply to the femoral head is thus critical. For example, in acetabular fracture, vascularization of the femoral head often cannot be assessed pre-operatively and a validated intra-operative prediction of poor femoral head prognosis would be helpful for the surgeon.

Two published studies described a technique to evaluate femoral head blood flow and perfusion [12–14], which consists of observing the blood spilling out from a drill hole in the femoral head, carried out immediately after dislocation. However, correlation between bleeding and subsequent AVN has not been demonstrated [14]. The aim of this study was to evaluate the correlation between the ‘bleeding sign’ and AVN in surgical hip dislocation for elective disease and for acetabular fractures.

## MATERIALS AND METHODS

Patients treated with surgical dislocation of the hip between February 2008 and June 2013 in a referral center for hip surgery were included in this prospective study. Forty-four patients with selected acetabular fractures, SCFE, and femoral head deformity were enrolled. Patients treated with surgical dislocation of the hip for femoroacetabular impingement were not included in this study because of the very low risk of AVN. All patients who met the indication for surgical dislocation were included. The authors’ indications for SCFE and acetabular fracture have already been described in other papers [2, 4]; surgical dislocation for severe femoral head deformity was performed according to Prof. Ganz’s indications [3]. Patients with follow-up shorter than 8 months were excluded. A hospital ethical committee approved the study and every patient provided informed consent before surgery.

Intra-operative assessment of the femoral head vascularity was performed through the ‘bleeding sign’, a procedure first described by Ganz [14]. This test consists of a 2.0-mm drill hole carried out on the non-weight-bearing area of the femoral head straight after its dislocation. The test was performed in all patients with the hip in adduction, flexion and external rotation. A positive bleeding test was taken to be an immediate appearance of active bleeding after drilling (see Supplementary Videos 1 and 2). The test was considered as negative if no bleeding occurs within the first 5 s after drill removal. In this study, we considered as negative all the dubious cases.

The development of AVN was considered the main outcome during follow-up. X-ray analysis and office visit interviews were planned at 40 days, 3 and 6 months, then yearly. Anteroposterior and lateral views of the most recent follow-up were reviewed for the purpose of this study. Patients were classified in the necrosis group if their images were classified as type II, III or IV according to Ficat classification [7]. Age, index diagnosis and length of follow-up were recorded for every patient.

The results were then dichotomized into no necrosis and necrosis groups to permit statistical evaluation. Bleeding sign prediction efficacy was analysed by application of Pearson’s Chi Square Test for setting sensitivity, specificity, positive and negative predictive values and accuracy. Accuracy was defined as the proportion of true results (both true positive and true negative) in the population.

## RESULTS

In this study, 44 patients who underwent surgical dislocation were enrolled (Table I). No patients were lost during follow-up. Mean age was 25 years and mean follow-up was 36 months (range 10 to 71 months). Thirty-eight out of 44 presented a positive intra-operative bleeding sign. On the contrary, six out of 44 demonstrated no bleeding after drilling of the femoral head. The prognostic ability of a positive intra-operative bleeding test is shown in Table II.

**Table I.** Population data SCFE

Patient	Age (years)	Follow-up (months)	Diagnosis	Bleeding sign	Signs of necrosis
1	15	71	SCFE	Positive	negative
2	36	71	Severe head deformity	Negative	positive
3	15	70	SCFE	Positive	negative
4	28	69	Acetabular fracture	Positive	negative
5	18	67	SCFE	Positive	negative
6	24	61	SCFE	Positive	negative
7	28	61	Acetabular fracture	Positive	negative
8	20	59	SCFE	Positive	negative
9	18	58	SCFE	Positive	negative
10	21	57	SCFE	Positive	Positive
11	41	57	Acetabular fracture	Positive	negative
12	20	54	SCFE	Positive	negative
13	17	51	SCFE	Positive	negative
14	18	51	SCFE	Positive	negative
15	19	50	Severe head deformity	Positive	negative
16	16	49	SCFE	Positive	negative
17	17	45	SCFE	Positive	negative
18	46	40	Acetabular fracture	Positive	negative
19	25	39	Severe head deformity	Positive	negative
20	29	36	Acetabular fracture	Positive	negative
21	14	34	SCFE	Positive	negative
22	31	34	Acetabular fracture	Positive	negative
23	57	32	Acetabular fracture	Positive	negative
24	46	26	Acetabular fracture	Positive	negative
25	16	25	SCFE	Positive	negative
26	15	24	SCFE	Positive	negative
27	16	24	SCFE	Positive	negative
28	13	23	SCFE	Positive	negative
29	16	21	SCFE	negative	Positive
30	14	20	SCFE	Positive	negative
31	33	19	Acetabular fracture	Positive	negative
32	15	18	SCFE	Positive	negative
33	14	17	SCFE	Positive	negative
34	45	17	Acetabular fracture	negative	negative
35	58	17	Acetabular fracture	Positive	negative
36	16	16	SCFE	Positive	negative
37	14	16	Severe head deformity	negative	Positive
38	13	13	Severe head deformity	Positive	negative
39	47	13	Acetabular fracture	negative	Positive
40	46	13	Acetabular fracture	Positive	negative
41	30	12	Acetabular fracture	Positive	negative
42	16	10	SCFE	Positive	negative
43	10	10	Severe head deformity	negative	Positive
44	42	10	Acetabular fracture	Positive	Positive

**Table II.** Cross table

Bleeding sign	No necrosis	Necrosis
Positive	37	1
Negative	1	5

Sensitivity for the ‘bleeding sign’ was 97%, specificity was 83%, positive predictive value was 97%, negative predictive value was 83% and accuracy was 95%. Pearson’s Chi Square Test of the association between AVN and positive/negative bleeding sign showed a statistically significant *P*-value (<0.001).

## DISCUSSION

According to the presented results, intra-operative bleeding sign is a reliable indicator of good femoral head prognosis after surgical dislocation of the hip. This achievement was also confirmed for patients with acetabular fractures.

Our study has some limitations. It was a single-center study with a relative small cohort of patients. We acknowledge also the limitation of this test: it is a non-instrumental analysis of the intra-operative femoral head blood flow but other authors have already described the scientific background of the proposed test [6, 13]. Furthermore, the choice of 5 s deadline is arbitrary and not based on scientific bases, and dubious cases are possible.

We based the test on a technique first described in late 1990s, when it was used to evaluate the vitality of the femoral head after neck fracture [13]. Ganz himself re-adapted the head drilling as a routine procedure for his 213 surgical hip dislocation for elective patients, in order to monitor femoral head vessel integrity during surgery [6]. No cases of AVN were reported in his study but AVN was described after surgical dislocation in SCFE patients and in acetabular fractures [2, 4]. Further techniques have been described for a more accurate intra-operative femoral head evaluation, employing electronic devices to monitor the pulse wave generated by blood flow into the spongious bone [12, 13]. However, these approaches do not substantially change our ability to predict femoral head vitality, although they help to scientifically support why bleeding sign is a convincing procedure [13].

We have found that intra-operative bleeding sign assessment is a valid indicator of femoral head vitality; the technique is fast, easy and affordable, and presents high sensitivity and specificity for AVN development. The prognostic power of the bleeding sign assessment performed at the beginning of surgery can be a determining factor for the type of surgery to perform. When no bleeding is observed, and so AVN is strongly expected, the surgeon may change his surgical plan. For example, the surgeon may opt for an acute non-conservative solution (such as total hip arthroplasty), but the test may also be useful if the surgeon prefers to continue with a conservative treatment. In trauma cases, a lighter and strategic fixation may be performed to simplify the possible subsequent non-conservative surgery, which is a likely event [9–11]. Screws may be strategically placed further from joint surfaces, in order to facilitate a potential subsequent non-conservative approach. In elective cases such as SCFE, sub-chondral wires may be placed further from the chondral bone to avoid future intra-articular penetration.

## CONCLUSION

Bleeding after femoral head drilling is a reliable test for AVN in patients who undergo a surgical hip dislocation.

## SUPPLEMENTARY DATA

Supplementary data are available at *Journal of Hip Preservation Surgery* online

## CONFLICT OF INTEREST STATEMENT

None declared.
